# Pulmonary neuroendocrine tumour-associated ectopic Cushing’s syndrome: diagnostic challenges and multidisciplinary management

**DOI:** 10.1530/EDM-25-0166

**Published:** 2026-03-26

**Authors:** Rubén D Carrasco B, Alba Juanes, Carmen Marrón, Mariña Veras, M Soledad Librizzi, María Calatayud

**Affiliations:** ^1^ Servicio de Endocrinología y Nutrición – Hospital Universitario 12 de Octubre, Madrid, Spain; ^2^ Servicio de Psiquiatría – Hospital Universitario 12 de Octubre, Madrid, Spain; ^3^ Servicio de Cirugía Torácica – Hospital Universitario 12 de Octubre, Madrid, Spain; ^4^ Servicio de Anatomía Patológica – Hospital Universitario 12 de Octubre, Madrid, Spain

**Keywords:** Cushing’s syndrome, pulmonary neuroendocrine tumour, ectopic ACTH secretion

## Abstract

**Summary:**

Ectopic adrenocorticotropic hormone (ACTH) secretion is a rare cause of Cushing’s syndrome (CS), often associated with neuroendocrine tumours (NETs). Early diagnosis can be difficult due to variable clinical manifestations and psychiatric symptoms that may obscure the underlying endocrine disorder. We report a case of severe ectopic ACTH-dependent Cushing’s syndrome secondary to a pulmonary NET, highlighting the importance of multidisciplinary management, advanced imaging with positron emission tomography/computed tomography with gallium-68-labelled DOTA-D-Phe-Tyr-octreotide (^68^Ga-DOTATOC PET-CT) and aggressive biochemical control to enable curative surgery and full clinical recovery.

**Learning points:**

- Ectopic ACTH secretion should be suspected in cases of severe, rapidly progressive hypercortisolism with marked hypokalaemia.
- Combined therapy with metyrapone and ketoconazole, along with hydrocortisone blockade and replacement, allows for rapid and safe cortisol control before surgery.
- ^68^Ga-DOTATOC PET-CT is essential for accurate localisation and staging of neuroendocrine tumours with ectopic ACTH production.
- Multidisciplinary management involving endocrinology, nuclear medicine, radiology, psychiatry, nutrition and thoracic surgery is crucial for successful outcomes.
- Early identification and aggressive management of metabolic and psychiatric complications are key to full recovery.

## Background

Cushing’s syndrome (CS) is a rare disorder, with approximately 10–15% of cases resulting from ectopic adrenocorticotropic hormone (ACTH) secretion (1). Its clinical spectrum ranges from mild presentations to severe hypercortisolism associated with high mortality. The underlying aetiology may involve well- or moderately differentiated neuroendocrine tumours (NETs) or, less frequently, poorly differentiated neuroendocrine carcinomas (NECs), which often complicates diagnosis and management (2). A multidisciplinary approach supported by advanced diagnostic techniques is therefore essential (1). This case illustrates the challenges of early diagnosis, comprehensive management, nutritional optimisation and continuous follow-up in ectopic ACTH-dependent Cushing’s syndrome.

## Case presentation

A 34-year-old man with a history of anxiety–depressive disorder and progressive obesity was admitted with rapidly progressive Fournier’s gangrene, requiring surgical debridement and secondary closure. During hospitalisation, he developed severe hypokalaemia (2.5 mEq/L) refractory to treatment, new-onset diabetes mellitus (glycated haemoglobin (HbA1c) 8.2%) requiring insulin therapy and fluctuating psychiatric symptoms, including mutism and refusal of treatment.

Physical examination revealed a rounded plethoric face, dorsocervical fat pad, central obesity, violaceous abdominal striae and marked proximal muscle weakness with generalised sarcopenia. These findings raised strong suspicion of Cushing’s syndrome (CS).

## Investigation

Endocrine evaluation confirmed hypercortisolism and ACTH dependency, with the following results:

- 24-h urinary free cortisol: 3,672 μg/24 h (reference range: 13–75 μg/24 h).
- Basal serum cortisol: 22.8 μg/dL (reference: 5–25 μg/dL).
- Plasma ACTH: 89.9 pg/mL (reference: 10–50 pg/mL).
- Serum cortisol after 1 mg dexamethasone suppression test: 35.7 μg/dL (expected <1.8 μg/dL).
- Nocturnal salivary cortisol: 3.17 μg/dL (reference <0.208 μg/dL).

Given the magnitude of hypercortisolism and the severity of clinical manifestations, CS due to ectopic ACTH secretion was strongly suspected.

**Figure 1:**
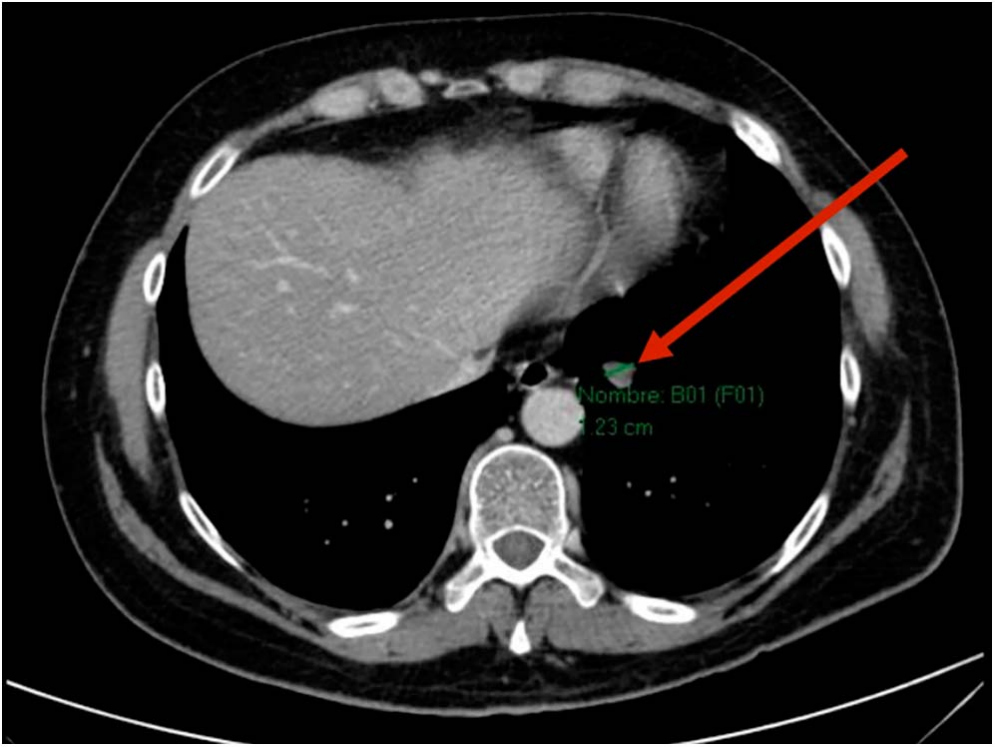
Thoraco-abdominal-pelvic CT scan. Axial view showing a 15 × 23 mm pulmonary nodular opacity at the base of the left lower lobe (arrow), without evidence of locoregional lymphadenopathy.

**Figure 2:**
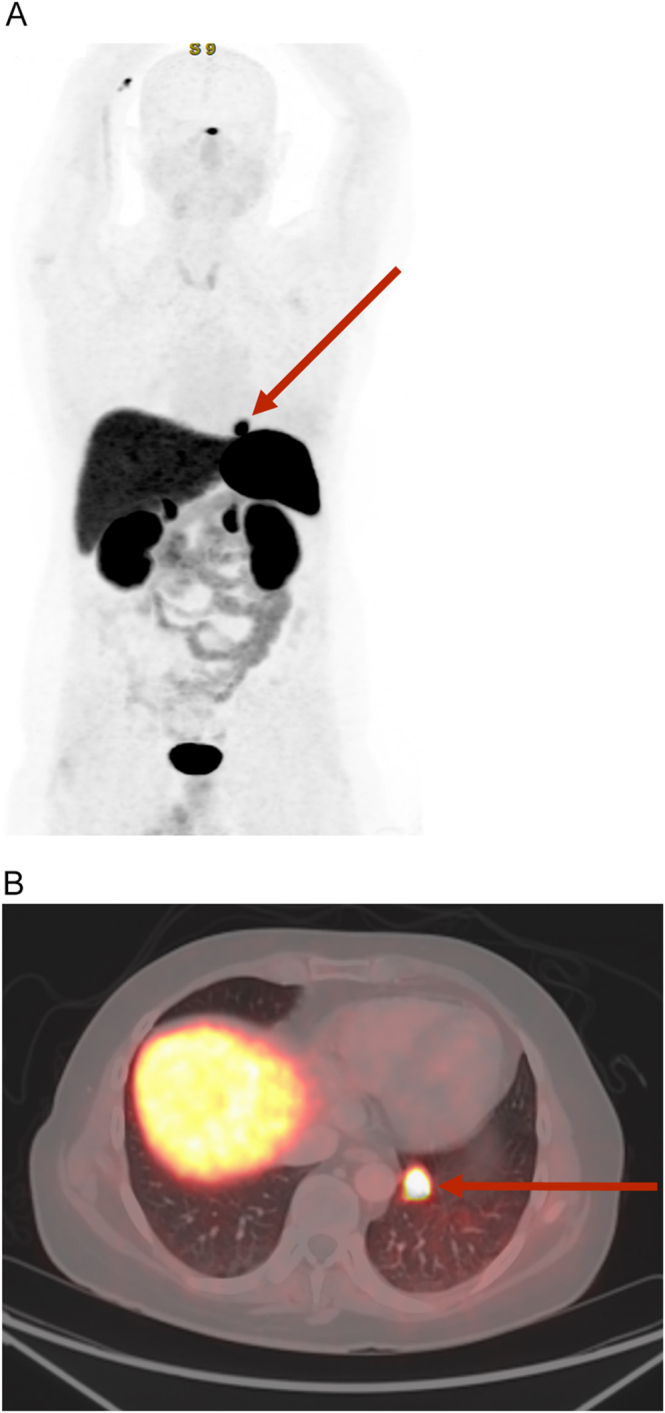
^68^Ga-DOTATOC-PET-CT scan. (A) Maximum intensity projection (MIP) PET image showing focal uptake in the left lower lobe (LII) (arrow). (B) Fused PET/CT image showing high uptake in the pulmonary nodule in the LII (SUVmax of 21.92, Krenning score of 3), consistent with somatostatin receptor overexpression.

To complete the aetiological study, a thoraco-abdominal-pelvic computed tomography (CT) scan revealed a 15 × 23 mm pulmonary nodular opacity at the base of the left lower lobe without locoregional adenopathy (Fig. 1). The lesion’s proximity to the diaphragmatic pleura and segmental vessels rendered percutaneous biopsy unfeasible due to the risk of pneumothorax and haemorrhage. Therefore, it was decided to complete the study with a ^68^Ga-DOTATOC PET-CT scan, which confirmed the presence of a pulmonary nodule with intense uptake (SUVmax of 21.9, Krenning score of 3), consistent with somatostatin receptor overexpression, with no evidence of additional tracer uptake (Fig. 2).

## Treatment

The patient was transferred to the intensive care unit for management of severe hypokalaemia and psychiatric monitoring, with limited cooperation. Medical therapy was initiated with metyrapone (up to 2,000 mg/24 h) and ketoconazole (up to 1,000 mg/24 h), with rapid escalation and a hydrocortisone blockade and replacement strategy. This combination regimen provided short-term control of hypercortisolism, ensuring normalisation of cortisol levels without compromising the patient’s adrenal function. The combination therapy was well tolerated, with no clinically significant adverse effects observed during rapid dose escalation. No treatment interruptions or dose reductions were required. Due to severe hypercortisolism-related diabetes mellitus, the patient required insulin therapy during hospitalisation, with progressive improvement in glycaemic control following biochemical control of cortisol excess.

Spironolactone (up to 200 mg/24 h) was added to manage hypokalaemia and hypertension. Thromboprophylaxis with low-molecular-weight heparin and antibiotic prophylaxis with trimethoprim–sulfamethoxazole were implemented.

The case was reviewed by a multidisciplinary tumour board, which recommended surgical resection of the pulmonary lesion once hypercortisolism was controlled. The patient received nutritional support with high-protein supplements and rehabilitation therapy prior to surgery. After achieving clinical stabilisation with progressive improvement in psychiatric symptoms and greater adherence to supportive therapies, a left lower lobectomy was performed.

## Outcome and follow-up

**Figure 3:**
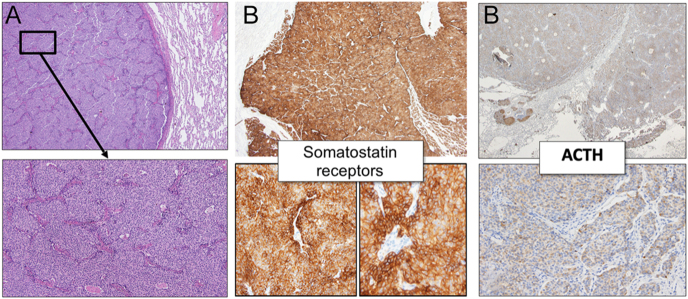
Histopathological findings of the resected pulmonary neuroendocrine tumour. (A) Microscopic image of the primary pulmonary lesion showing the typical architecture of a well-differentiated neuroendocrine tumour (H&E stain). (B) Immunohistochemical staining showing positivity for somatostatin receptors (SSTR), ACTH and other neuroendocrine markers (chromogranin, synaptophysin and INSM1).

Histopathological analysis confirmed the presence of a typical pulmonary neuroendocrine tumour (NET, WHO 2021 classification, grade 1) measuring 2 cm in maximum dimension, with clear surgical margins (protein tyrosine phosphatases 1B and N2 (pT1b N2)). Microscopic examination showed low mitotic activity (1 mitosis/mm^2^; ≤2 mitoses/2 mm^2^) and the absence of tumour necrosis, consistent with the diagnostic criteria for a typical carcinoid tumour. A supradiaphragmatic adenopathy showed lymph node metastasis of a typical grade 1 carcinoid NET with extracapsular extension. The Ki-67 proliferation index was 1.7% in the primary tumour and 2.1% in the lymph node metastasis. Immunohistochemistry studies were positive for TTF-1 (thyroid transcription factor 1), neuroendocrine markers (insulinoma-associated protein 1 (INSM1), chromogranin and synaptophysin), ACTH and somatostatin receptors (SSTR) (Fig. 3).

Surgery enabled discontinuation of medical therapy for hypercortisolism. The patient was transferred to a continuity-of-care facility, where Fournier’s gangrene was healed and sarcopenia was recovered. Surgery also enabled the complete resolution of fluctuating neuropsychiatric symptoms and the healing of Fournier’s gangrene.

The patient has been followed for 20 months after surgery, with sustained clinical and biochemical remission and no evidence of recurrence.

## Discussion

Ectopic ACTH secretion accounts for 10–15% of Cushing’s syndrome cases, with bronchial NETs being among the most frequent causes (1). Typical bronchial NETs usually appear as solitary, well-defined pulmonary nodules with high vascularisation and somatostatin receptor expression. The clinical presentation of ectopic ACTH secretion is often severe, characterised by rapid onset of marked hypercortisolism, profound hypokalaemia, metabolic decompensation, increased susceptibility to infection and neuropsychiatric manifestations (1). Psychiatric manifestations, as in this case, may obscure early diagnosis and complicate management. In our patient, approximately three years elapsed between the onset of nonspecific symptoms and the etiological diagnosis; however, once Cushing’s syndrome was clinically suspected, biochemical confirmation was achieved within three days, allowing prompt localisation and definitive surgical management.

**Figure 4:**
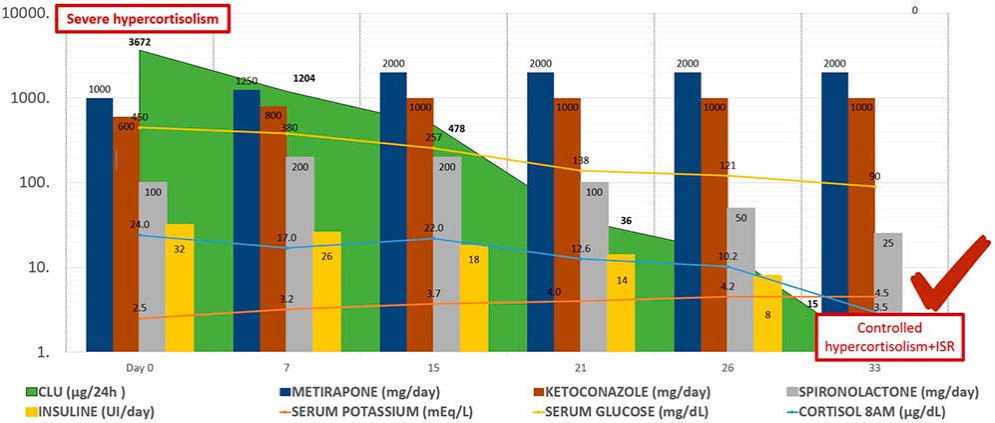
Biochemical response and therapeutic course during metyrapone–ketoconazole combination therapy. The green-shaded area represents 24-h urinary free cortisol (UFC, μg/24 h), showing progressive reduction from severe hypercortisolism to biochemical control. The shaded section marked ISR indicates the period of secondary adrenal insufficiency, when morning serum cortisol levels decreased to approximately 3.5 μg/dL, reflecting adequate suppression of adrenal function during block-and-replace therapy.

Rapid biochemical control is critical to reduce morbidity and prepare the patient for curative surgery (3). Combination therapy with steroidogenesis inhibitors (such as metyrapone and ketoconazole) and a hydrocortisone blockade–replacement approach provides effective short-term control of cortisol excess (4, 5) (Fig. 4). In our patient, the clinical response was optimised with this combination, while maintaining adequate cortisol levels to preserve critical metabolic functions and prevent adrenal crises.

Functional imaging with ^68^Ga-DOTATOC PET-CT is now considered the gold standard for localising NETs, due to its high sensitivity for somatostatin receptor expression (2, 6). In our case, it enabled precise localisation and staging, facilitating surgical planning. Surgical resection remains the treatment of choice for localised disease and can lead to biochemical remission in most cases. In our case, surgery allowed discontinuation of hypercortisolism therapy and complete clinical recovery.

Multidisciplinary collaboration between endocrinology, nuclear medicine, radiology, psychiatry, nutrition and thoracic surgery teams is fundamental to achieving optimal outcomes (1), as demonstrated in this case.

## Declaration of interest

The authors declare that there is no conflict of interest that could be perceived as prejudicing the impartiality of the study reported.

## Funding

Esteve Pharmaceuticals SA provided an unrestricted grant to assist in the publication of this case report. Esteve Pharmaceuticals was not involved in the preparation of the manuscript, or in the decision to publish the case report.

## Patient consent

Written informed consent for publication of their clinical details and clinical images was obtained from the patient. A signed copy of the consent form is collected in the patient’s archive.

## Author contribution statement

All authors contributed equally, and all reviewed and approved the final version of the manuscript.
